# *In Vivo* Assessment of Cold Adaptation in Insect Larvae by Magnetic Resonance Imaging and Magnetic Resonance Spectroscopy

**DOI:** 10.1371/journal.pone.0003826

**Published:** 2008-12-05

**Authors:** Daniel Mietchen, Bertram Manz, Frank Volke, Kenneth Storey

**Affiliations:** 1 Magnetic Resonance Group, Fraunhofer Institute for Biomedical Engineering (IBMT), St. Ingbert, Germany; 2 Faculty of Physics and Mechatronics, University of the Saarland, Saarbrücken, Germany; 3 Department of Psychiatry, Friedrich-Schiller University Jena, Jena, Germany; 4 Institute of Biochemistry, Carleton University, Ottawa, Ontario, Canada; University of Western Ontario, Canada

## Abstract

**Background:**

Temperatures below the freezing point of water and the ensuing ice crystal formation pose serious challenges to cell structure and function. Consequently, species living in seasonally cold environments have evolved a multitude of strategies to reorganize their cellular architecture and metabolism, and the underlying mechanisms are crucial to our understanding of life. In multicellular organisms, and poikilotherm animals in particular, our knowledge about these processes is almost exclusively due to invasive studies, thereby limiting the range of conclusions that can be drawn about intact living systems.

**Methodology:**

Given that non-destructive techniques like ^1^H Magnetic Resonance (MR) imaging and spectroscopy have proven useful for *in vivo* investigations of a wide range of biological systems, we aimed at evaluating their potential to observe cold adaptations in living insect larvae. Specifically, we chose two cold-hardy insect species that frequently serve as cryobiological model systems–the freeze-avoiding gall moth *Epiblema scudderiana* and the freeze-tolerant gall fly *Eurosta solidaginis*.

**Results:**

*In vivo* MR images were acquired from autumn-collected larvae at temperatures between 0°C and about −70°C and at spatial resolutions down to 27 µm. These images revealed three-dimensional (3D) larval anatomy at a level of detail currently not in reach of other *in vivo* techniques. Furthermore, they allowed visualization of the 3D distribution of the remaining liquid water and of the endogenous cryoprotectants at subzero temperatures, and temperature-weighted images of these distributions could be derived. Finally, individual fat body cells and their nuclei could be identified in intact frozen *Eurosta* larvae.

**Conclusions:**

These findings suggest that high resolution MR techniques provide for interesting methodological options in comparative cryobiological investigations, especially *in vivo*.

## Introduction

Cold hardiness–the ability to withstand temperatures below the freezing point of water–has been found in a broad variety of poikilothermic species ranging from prokaryotes to plants to nematodes to insects and fish, and it also occasionally occurs in amphibians or reptiles [1]. Insects clearly belong to the taxa whose cold hardiness has been investigated most thoroughly, in part due to commercial interests in killing pest species in agriculture and forestry [2] or in managing valuable species, such as stocks of genetic model organisms like the fruitfly *Drosophila melanogaster* [3].

Methodologically, whole-body extracts have been the most popular choice for studies of cryoprotectant synthesis and other aspects of intermediary metabolism, usually performed using quantitative organochemical techniques as well as biochemical methods like enzyme assays and kinetic analyses [4], [5]. The presence of antifreeze proteins or ice nucleating proteins in hemolymph is analyzed by various osmometric techniques or differential scanning calorimetry [6], [7], and electron and optical microscopic techniques have been used in some studies to elucidate ultrastructural details of cryodamage [8], [9].

What all these different approaches have in common is that they do not allow for *in vivo* observations, albeit the latter are generally more informative and now commonplace in many branches of the biosciences. Magnetic Resonance (MR) techniques, in particular, have found many applications in the life sciences, especially whenever liquid water was of interest (e.g. [10]–[14]). Since this is the case in cryoprotection, too, we designed the present study to assess the potential of high resolution MR imaging for cryobiological investigations.

### Cryobiological model organisms

The animals we chose for our study were the larvae of two long-standing model species for insect cold hardiness–the freeze-avoiding gall moth *Epiblema scudderiana* and the freeze-tolerant gall fly *Eurosta solidaginis*. They share the same habitat (stem galls on goldenrod plants, genus *Solidago*) but use different overwintering strategies [15]. *Epiblema* larvae accumulate huge amounts of glycerol, up to 20% of their wet body mass, during autumn cold-hardening [16]. This–combined with the probable presence of ice-structuring proteins [17] in this species, elimination of ice nucleators from the body, and partial dehydration [16], [18]–allows deep supercooling of body fluids down to approximately −38°C, which is about 10°C below the lowest temperatures that *Epiblema* larvae typically experience during winter.

*Eurosta* larvae also synthesize cryoprotectants in response to low temperature cues as the autumn progresses. They achieve this by mobilizing large glycogen stores that are built up during the feeding season in summer. This species uses a two-component system with glycerol synthesis triggered when ambient temperatures fall below about +15°C, whereas sorbitol production is stimulated below +5°C. Likewise, membrane stabilizers such as the disaccharide trehalose and the amino acid proline are produced during the period of cold hardening. During autumn, the larvae also elevate the supercooling point of body fluids to about −8°C, so that freezing can be triggered at a relatively high subzero temperature that allows proper ice management and metabolic adjustments upon the onset of freezing temperatures [4]. Prepared this way, *Eurosta* larvae can survive in a frozen state down to at least −30°C in southern Canada.

### MR principle

The concept of Nuclear Magnetic Resonance (commonly abbreviated NMR or MR) has repeatedly found comprehensive treatment elsewhere–see, e.g., [19] or [20] for spectroscopy and [13] or [12] for imaging–and will therefore only briefly be sketched here. In short, MR describes the absorption of electromagnetic energy by a subpopulation of atomic nuclei in an external static magnetic field when irradiated at an isotope-specific resonance frequency directly proportional to the local magnetic field strength (or magnetic flux density, to be precise, but the two units are often used interchangeably in this context, since they are proportional to each other). When the absorbed energy is released upon return to the thermal equilibrium, an inductive signal (with the relaxation times *T*_1_ and *T*_2_ parallel or perpendicular to the axis of the main magnetic field, respectively) can be observed.

In liquids, the reorientation of the dipolar interaction is usually in the fast motion regime, where both *T*_1_ and *T*_2_ are on the order of several 100 s of milliseconds or seconds. In solids, the rate of this reorientation is dramatically reduced and therefore in the slow motion regime ([20], [13]). As a consequence, the longitudinal MR relaxation time *T*_1_ of water ice is similar to the *T*_1_ of liquid water but the transverse relaxation time *T*_2_ is about five to six orders of magnitude lower than in liquid water of the same composition [21]. With the exponential relationship between the MR signal and *T*_2_ [19], this translates (under identical acquisition schemes) into an enormous signal loss upon freezing and a corresponding signal increase upon thawing, which provide a good theoretical basis for the experimental observation of these processes.

The local field strength that an atomic nucleus within a sample of interest experiences in the MR magnet is a function of both the static magnetic field (9.4 T in our case) and the molecular neighbourhood in which that nucleus (say, ^1^H) is embedded (say, an OH or a CH_3_ group). The resonance frequencies of a given type of nucleus (e.g. 400 MHz for ^1^H at 9.4 T) thus vary slightly (in some parts per million, or ppm) with its chemical environment–a phenomenon known as chemical shift that forms the basis for MR spectroscopy (MRS). Other than by electron shielding, the local field strength can be manipulated through magnetic fields supplementary to the static magnetic field. If the field strengths of these are space-dependent, the resonant frequency becomes space-dependent, too, which allows for the generation of spatial representations of the sample's MR-accessible structures. This approach is known as MR imaging (MRI). Following conventions in optical microscopy, MRI at spatial resolutions below the spatial resolution limit of human vision (about 100 µm) is often also referred to as MR microscopy, or MRM [12], [13], [22].

Short *T*_2_ relaxation times, i.e. quick signal decay which generally indicates that many of the MR-visible nuclei are restricted in their molecular mobility, result in broad peaks in MR spectra. If an originally liquid sample with several well-separated MR peaks were to become entirely solid, the different peaks would broaden, start to overlap and finally form one unspecific peak spanning the whole spectrum, even though the underlying processes are not necessarily identical for different molecular species: while all peaks broaden due to relaxation effects, the OH peak in the ^1^H spectrum of aqueous solutions experiences additional (exchange) broadening due to exchange with OH protons from the surrounding water.

In MR imaging, the time needed for signal acquisition generally increases with the number of image pixels that the resulting image is to contain in a given direction. The quick *T*_2_ decay hence seriously impedes the acquisition of high resolution MR images from solids, and signal contributions can in general terms only be expected from the narrowest peaks of the spectrum at the given temperature.

Spatial and chemical information can be combined in MR experiments, in two major fashions: Localized spectroscopy [23] allows the acquisition of spectral information from well-defined volume elements (or voxels) within the sample, whereas spectral imaging techniques (known as Chemical Shift-Selective Imaging, abbreviated CHESS, CSSI or CSI; reviewed in [24]) map the spectral contributions from nuclei in selected chemical environments (e.g. ^1^H in water or aliphatic groups) along the selected spatial dimensions of the sample.

### Previous MR studies relevant to cryobiology

The first applications of MR to frozen samples of biological relevance was the determination of brine content in sea ice by comparing the integral MRS signal intensity within a sea ice sample and a solution of known composition that was obtained by melting a sea ice sample [25].

MRS has since found multiple applications related to cryobiology, ranging from ^31^P analysis of adenylate energy metabolism during freezing [26], to ^13^C studies of lipids and carbohydrates [27] to ^1^H studies of glucose metabolism and transamination after thawing [28] as well as of water content in plants [29], biocatalytic model systems [30] or food [31] at subzero temperatures.

Brine content again was the dominating feature in the first ^1^H MR images of salt-water ice mimicking the mineral composition of sea water [32], and of sea ice cores ([33], acquired at geomagnetic field strength in Antarctica). Furthermore, the diffusivity of the brine phase in intact sea ice cores was measured [34], the previously reported temperature dependence of brine content [35] could be correlated with changes in brine pocket size by MR imaging of sea ice [36], and the freezing process was followed in a sea ice model system [37].

Apart from monitoring brine content, ^1^H MR imaging has been used to observe pore structure in permafrost samples [38], to follow ice nucleation in single drops of cryoprotective solutions [39], for temperature mapping and cryodamage assessment during cryosurgery [40], [41], and for the monitoring of freezing and thawing in food [42], cold-hardy plants [43], [44] and animals such as frogs and turtles [45], [46].

CHESS imaging has previously been used to investigate insect larval development (e.g. [47]) in a non-cryobiological context, and technological developments in the MR field have recently seen *in vivo* spatial resolution in non-frozen samples reach the size range relevant for entomological [48] and subcellular investigations [49], [50].

The present study combined these two fields of investigation by demonstrating the feasibility of high-resolution MR imaging of insect larvae in a cryobiological context.

## Methods

### Insect collection and preparation

*Solidago* galls containing larvae of *Epiblema scudderiana* and *Eurosta solidaginis* were collected from fields around Ottawa, Canada in late October 2005. Galls were placed in cloth bags and kept outdoors until mid-December where they experienced ambient temperatures which included subzero temperatures most nights (lowest values being −10°C to −12°C on some nights). Previous studies have shown that glycerol levels are maximal in both species by late November, whereas sorbitol in *Eurosta* reaches at least 50% of the midwinter levels by this time [16], [26]. In mid-December, galls were shipped to Germany in insulated containers with cold packs to maintain temperature at 0–5°C and were stored in the lab at 1°C until experimentation.

**Figure 1:**
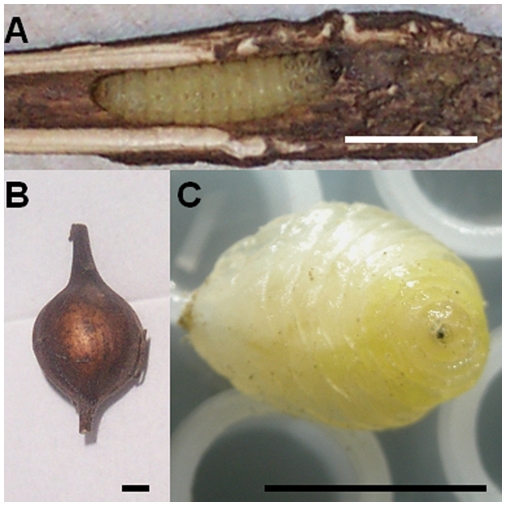
Galls and larvae used in this experiment. (A) Photograph of an *Epiblema scudderiana* larva (head is right) in its gall, taken after the experiment. The gall was left intact throughout the experiments and only cut open afterwards to confirm the position during the NMR experiments. Normal position both in nature and in the magnet is upright. (B) Photograph of an *Eurosta solidaginis* gall before the start of the experiment. The insect was removed from the gall for the sake of increased spatial resolution. (C) Photograph taken after the experiment, indicating the positioning on the horizontal microwell plate. Note the yellowish appearance due to glycerol. All scale bars in this figure represent 5 mm.

*Epiblema* larvae were imaged while still in their galls (cf. Fig. 1A). The galls induced in *Solidago* stems by *Eurosta solidaginis* are much larger, which complicates high resolution imaging of the larvae themselves. For this reason, *Eurosta* larvae were removed from their galls (cf. Fig. 1B) and positioned on cryowell plates that are routinely used for cryobiological applications at Fraunhofer-IBMT [51] (cf. Fig. 1C).

For each species, the imaging and cooling protocols were tested with one individual in a pilot experiment, and all data shown here are from another individual freshly obtained from the cooling chamber directly prior to the experiment.

### Magnetic Resonance

#### MR parameters

**Figure 2:**
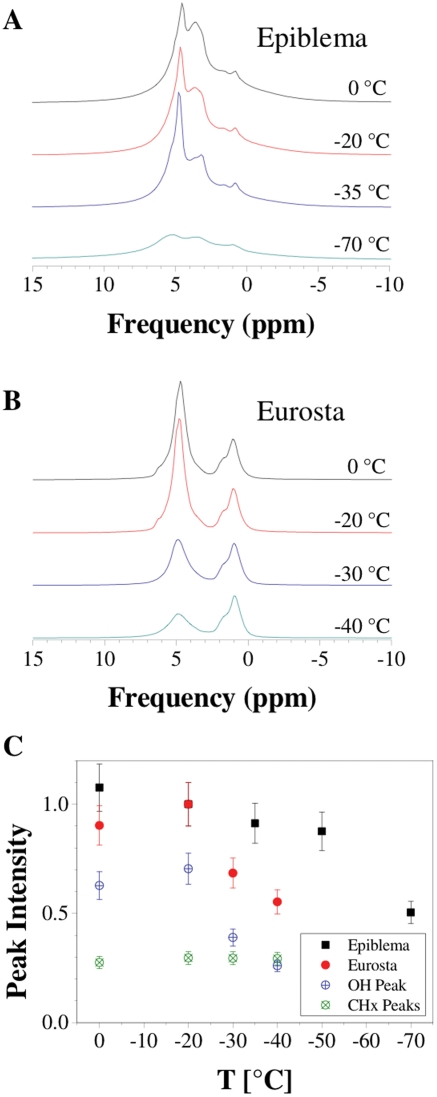
^1^H NMR spectra of insect larvae at different temperatures (intensity in arbitrary units but on scale across temperatures). A: *Epiblema*, B: *Eurosta*. The broad peak referenced to 4.7 ppm represents hydroxyl groups (in water, glycerol and sorbitol), whereas hydrocarbon groups from glycerol and sorbitol are located around 3.6 ppm, and otherhydrocarbon groups, e.g. from lipids, around 1–2 ppm. Note that glycerol is present in both species, sorbitol only in *Eurosta*. For acquisition parameters in the images presented in this paper, see Methods section. C: Freezing trajectory, as extracted from the spectra above by integrating over the whole spectrum (*Epiblema*, black squares) or individual peaks (*Eurosta*, circles with upright cross for OH peak, circles with oblique cross for CH_x_ peak, red circles for whole spectrum) at the given temperature. Signal intensity in each species was normalized to the respective value measured at −20°C. Note that the CH_x_ peak integral does not change with temperature, while the OH peak integral does, thereby being responsible for the changes seen in the whole-spectrum trajectory in *Eurosta*. Most (if not all) of the signal loss observed in the *Epiblema* trajectory are presumably also due to the freezing of water but these contributions could not be reliably separated, since the peaks overlap in the spectrum (cf. A).

The MR images and spectra described in here were acquired on a Bruker Avance 400 NMR spectrometer (Bruker, Rheinstetten, Germany) with standard Micro2.5 microimaging system, operating at a ^1^H resonance frequency of 400 MHz. For the *Epiblema* larva, typical proton density images were recorded using a 3D spin-echo imaging pulse sequence with a field of view of 12×12×12 mm^3^, a matrix size of 256^3^ voxels, an echo time *T_E_* = 2.2 ms, a repetition time *T_R_* = 0.9 s, resulting in an isotropic voxel resolution of 47 µm. The 3D proton density images for the *Eurosta* larva were acquired with a field of view of 7×7×3.5 mm^3^, a matrix size of 256×256×128 voxels, an echo time *T_E_* = 1.8 ms, a repetition time *T_R_* = 1 s, resulting in an isotropic voxel resolution of 27 µm. CHESS imaging was achieved with selective excitation of the water or lipid resonances (cf. Fig. 2) by means of a 4 ms long sinc-shaped excitation pulse with the frequency of the water or lipid resonance [23]. Due to this long excitation pulse, the echo time had to be increased to 4.7 ms, resulting in a large signal loss compared to the non-selective images as a consequence of *T_2_* relaxation. Therefore, the matrix size was reduced to 64×64×32 voxels at temperatures below −20°C, resulting in an isotropic voxel resolution of 110 µm. The repetition time *T_R_* was then set to 0.5 s.

The images were processed and visualized with the help of ImageJ (developed at the National Institutes of Health and available online via http://rsb.info.nih.gov/ij). Three-dimensional models were calculated as isosurfaces from the MR images using the software package Amira (Mercury Computer Systems, Merignac, France).

### Temperature control

Temperature regulation was achieved using a Bruker variable temperature unit BVT3000. Liquid nitrogen was evaporated in a closed 30 l dewar using a small heater. The cold nitrogen gas was then heated up by the temperature controller before streaming around the glass tube containing the sample. The temperature sensor (copper-constantan thermocouple) had to be placed approximately 4 cm away from the sample in order to avoid image distortions due to the metal parts. At the sample location, the temperature was calibrated via the line splitting between the methyl and hydroxyl groups in a 100% methanol sample [52]. Temperature changes were always administered at a rate of 1 K/min, and once the target temperature was reached, the sample was allowed at least one hour for thermal equilibration. Then, temperature-dependent MR parameters were adjusted, and the temperature was kept constant for each acquisition of an image or spectrum.

## Results

### MR spectroscopy

Fig. 2 shows ^1^H MR spectra of an *Epiblema* larva (A) and an *Eurosta* larva (B), acquired at different freezing temperatures, while the signal changes with temperature are indicated in the diagram at the bottom. The two broad peaks in the spectra can be assigned (following [53]) to hydroxyl groups from water (4.7 ppm) as well as glycerol and sorbitol (4.5 ppm) and to hydrocarbon groups from glycerol and sorbitol (around 3.5 ppm) as well as other sources, e.g. lipids (around 1–2 ppm). With decreasing temperature, peak broadening and an overall loss in signal intensity become apparant (cf. section on the MR principle).

While the peaks in the *Epiblema* spectra (Fig. 2A) exhibit a degree of overlapping that does not allow for a detailed separate analysis, the hydroxyl and hydrocarbon peaks in *Eurosta* (Fig. 2B) are well separated in the spectra. Therefore, the relative portions of these compounds can be estimated from the integrals of the respective peaks: Taking the integral of the hydroxyl peak at −20°C as a reference, the hydroxyl peak at −40°C reaches about 35% thereof, while the hydrocarbon peaks at −20°C and −40°C each stand at about 40% of that reference value (cf. Fig. 2C). Given that there are no hydrocarbon groups in water and that both glycerol and sorbitol have equal numbers of hydroxyl and hydrocarbon groups, this decline of the hydroxyl peak integral relative to the hydrocarbon peak integral clearly indicates that the system has lost liquid water due to freezing.

This effect can also be quantified, at least approximately: although a single or few layers of water molecules can remain in a liquid phase even below the homogeneous nucleation temperature as a result of surface interactions with non-water molecules [30], [54], [55], it can be assumed, for simplicity, that the hydroxyl signal at −40°C essentially stems from glycerol and sorbitol. The 65% signal loss of the hydroxyl peak between −20°C and −40°C can thus be attributed to the freezing of water. This also means that liquid water accounts for 65% of the hydroxyl signal at −20°C.

The freezing trajectory (Fig. 2C) of the hydrocarbon peak in the *Eurosta* spectra, on the other hand, remains constant within the inspected temperature range, reflecting the absence of a phase transition in the hydrocarbons (which include non-glycerol and non-sorbitol contributions, e.g. from membrane lipids). So it cannot be used for the direct observation of freezing process but it can serve as an internal reference to the hydroxyl peak.

As for *Epiblema*, though the peaks in the spectra could not be separated, the freezing trajectory extracted from the spectra also clearly shows a signal loss at lower temperature, attributable to freezing (Fig. 2C).

### MR imaging

**Figure 3:**
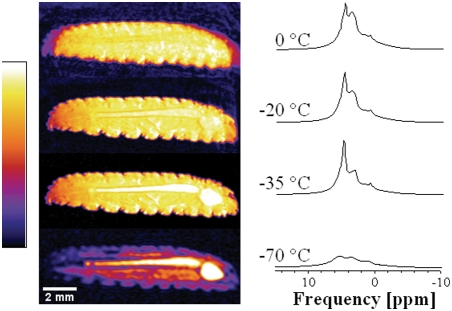
Correspondence between NMR images and spectra: ^1^H NMR images and corresponding NMR spectra of an *Epiblema* larva at different temperatures. Relative signal intensity (from black = low to white = high; identical for all MR images in this paper, even though absolute intensities vary considerably) is given in arbitrary units but on scale across temperatures. Image resolution: 47 µm (isotropic). The images were rotated by 90° for better presentation–positioning of the larva was with head up (right), tail down (now left). The spectra are the same as in Fig. 2A and repeated here to facilitate interpretation of the images. Note the image blurring at 0°C and −20°C, hinting at head and tail motion of the larva. The complete slice series of these four 3D images are given as Movie S1. This video also shows the signal contributions from the *Solidago twig*, which exhibit a different temperature dependence than the signal from the larva.

Fig. 3 shows slices from a 3D ^1^H MR image (left) and ^1^H MR spectra (right), acquired at the indicated temperatures, of the *Epiblema* larva in an orientation similar to the photograph in Fig. 1A. When the images and spectra were recorded, the *Solidago* gall was still intact, albeit not visible in the MR images on this contrast scale.

With decreasing temperature, the signal intensity in the images is decreasing too (due to temperature-induced peak broadening, as explained above), though inhomogeneously (due to spatially inhomogeneous distribution of water and cryoprotectant). The high overall signal intensity in the images acquired at the higher temperatures clearly reveals that the larva is not frozen and instead still in a supercooled state. At temperatures above −35°C, the contours of the anterior segments appear slightly blurred due to a movement artifact that testifies to the vitality of the animal. However, after being cooled down to temperatures below its supercooling point, the *Epiblema* larva did not revive, which fits with its classification as a freeze-avoiding species.

### Spectral imaging

**Figure 4:**
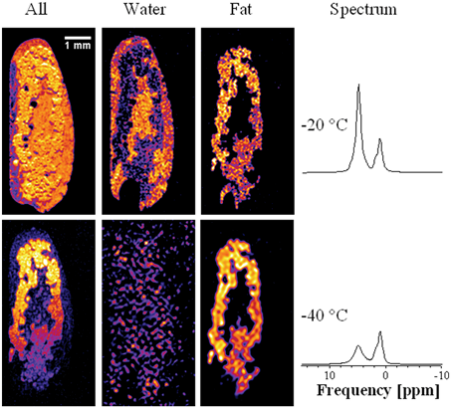
Imaging the three-dimensional distribution of water and cryoprotectant in a living frozen organism: ^1^H NMR images and corresponding NMR spectra of an *Eurosta* larva at different temperatures (intensity in arbitrary units but on scale across temperatures). All images have been rotated by 90°for ease of display (direction of gravity was from right to left). The images labeled “All” were acquired by using the whole spectrum (at a resolution of 27 µm), whereas the “Water” and “Fat” images only represent contributions from the hydroxyl peak at 4.7 ppm and the hydrocarbon peak at around 1 ppm, respectively (acquired at a resolution of 27 µm at −20°C and of 110 µm at −40°C; the latter were zero-filled to facilitate comparison across temperatures). The overall signal intensity of the water and fat images at a given temperature do not add up to the intensity of the whole-spectrum image because the CHESS sequences required longer echo times (for details, see methods section). The spectra are repeated from Fig. 2B to facilitate interpretation of the images. Note the single cells visible (with their nuclei) in all three images at −20°C and particularly in the whole-spectrum image at −40°C. Shape distortions with respect to normal anatomy of *Eurosta* larvae are due to the positioning on the cryowells, as indicated in Fig. 1C, while seemingly morphological differences between the images also reflect the rescaling of the images, or movements of the animal. The complete slice series of these six 3D images are presented separately by temperature (−20°C or −40°C) and spectral coverage (All/Water/Fat) in Movies S2, S3, S4, S5, S6 and S7: At −20°C, Movie S2 covers the whole spectrum and Movie S3 and Movie S4 the water and fat image, respectively. At −40°C, Movie S5 covers the whole spectrum and Movie S6 and Movie S7 the water and hydrocarbon images, respectively. Image orientation in the movies reflects the horizontal positioning of the larva during the experiment (cf. Fig. 1C).

Fig. 4 depicts 3D ^1^H MR images and spectra acquired from an *Eurosta* larva at two different temperatures. The figure also demonstrates how CHESS imaging (for details, see Methods) can reveal details about the 3D distribution of different chemicals: Image slices labeled “All” were taken from 3D images acquired using chemical information from the whole spectrum, whereas the slices labeled “Water” and “Fat”, respectively, were taken from 3D images that used spectral information from either the water or hydrocarbon peaks.

Interestingly, the water and hydrocarbon images (cf. Fig. 4) were very distinct and almost complementary to each other, thereby highlighting the potential of CHESS imaging to assist investigations into the 3D distribution of water and cryoprotectants. While the spectrum acquired at −40°C already indicates that most of the water in the larva was frozen, the CHESS images reveal that freezing does not occur homogeneously across the organism.

Individual cells are hard to identify in regions where strong signal losses (and thus freezing) do occur but, interestingly, a number of large cells (termed fat cells–those with a higher signal intensity in the hydrocarbon images, indicating larger quantities of cryoprotectant) clearly remain non-frozen and even allow their nuclei to be discerned (see dark spots therein), particularly in the whole-spectrum images.

MR techniques have a two decade-long history of single-cell imaging (cf. [56]), and *in vivo* MR images of single cells have been acquired before (for recent reviews, see [22], [57]) but to our knowledge, these fat cells represent the first instance of individual cells having been imaged *in vivo* inside an opaque multicellular organism in a frozen state (corresponding optical images have been obtained by [58] in a transparent Antarctic worm). That the larva were still alive during the experiment was confirmed by the motion artefacts (cf. Fig. 4) and by the observation–in line with the classification of *Eurosta solidaginis* as a freeze-tolerant species–that they successfully revived and moved normally after thawing.

**Figure 5:**
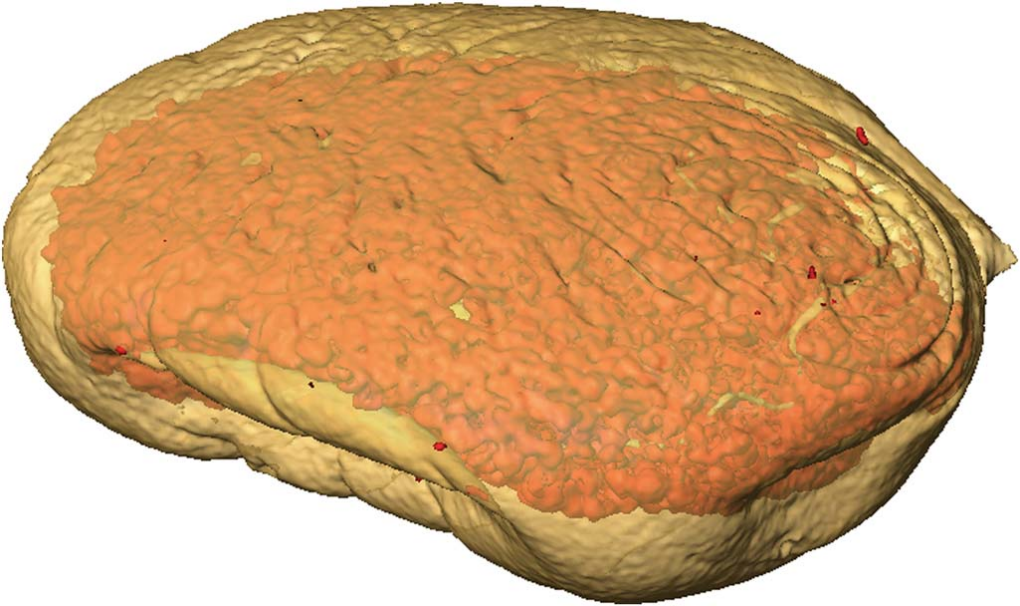
Three-dimensional modeling: 3D model of a *Eurosta* larva, based on ^1^H NMR water and fat images (cf. Fig. 3) obtained at −20°C. The water-based 3D model is depicted in semi-transparent brown and the model based on the fat signal in red. An animation of this chemical shift-coded composite 3D model is supplied as the supplementary video file Movie S8.

**Figure 6:**
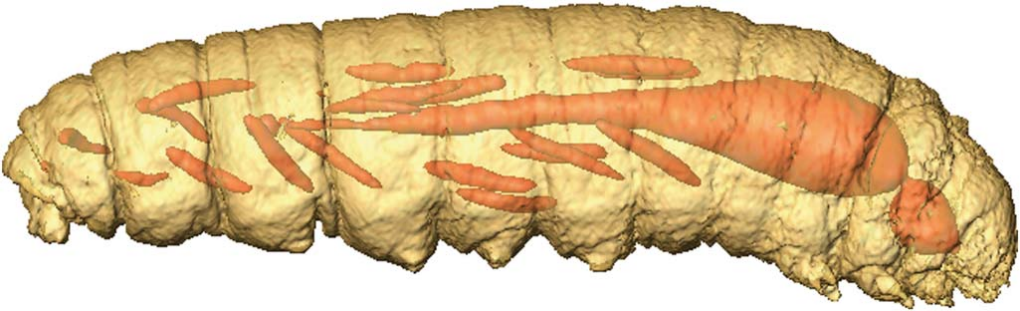
Temperature-weighted imaging: 3D model of an *Epiblema* larva, based on ^1^H NMR whole-spectrum images (cf. Fig. 4) obtained at −35°C (brown) and −70°C (red). An animation of this temperature-coded composite 3D model is supplied as the supplementary video file Movie S9.

As the data acquired from the larvae had spatial, spectral and temperature dimensions for both species, a number of possibilities exist to visualize them. This is illustrated in Fig. 5 and Fig. 6 which emphasize two different aspects of the methodology: Fig. 5 shows the 3D distribution of water and hydrocarbons in the *Eurosta* larva at −20°C, thereby highlighting spectral (CHESS) imaging. Fig. 6 differentiates between the whole-spectrum 3D ^1^H MR images acquired at −35°C from the *Epiblema* larva and the corresponding images at −70°C, thereby providing for an example of temperature-coded imaging. We are not aware of any other method that would allow to generate temperature-weighted three-dimensional images of intact organisms, though this tool for the quantification of temperature effects would certainly be of use for many a cryobiological investigation (e.g. on possible hysteresis effects in repeated freeze-thaw cycles, or for the monitoring of the interaction of tissue with markers or transgrafts) as well as in teaching cryobiology.

## Discussion

The experiments described in this paper have presented a new cryobiological tool by showing that three-dimensional microscopic MR images can be acquired from intact insect larvae under cryobiological conditions. The properties observable in these image sets include anatomical structures of the larvae down to the cellular level as well as larval chemical composition, or temperature-dependent changes thereof.

This opens the door for further MR-based cryobiological investigations: It is known, for example, that non-frozen fat body cells of overwintering Eurosta larvae have a water content of only about 35% ([59]). The images in Fig. 4, then, raise questions about the distribution of this little amount of water (liquid or frozen) and cryoprotectant between extracellular and intracellular compartments at different temperatures. Such issues are hard to address experimentally in vivo (for previous work, see [60] on cells and [58] in intact organisms) but the MR signal, and therefore the MR image contrast, is a reflection of the molecular interactions of molecular species in different chemical environments. Thus, water and hydrocarbon signals can be separated on the basis of MR spectra, and signal contributions from cellular and extracellular structures with MR-based diffusion measurements (as in [61]). Consequently, MR microimaging studies can be a valuable aid in gaining a deeper understanding of cold adaptations at the cellular level.

Furthermore, MR microimaging could be used to develop and test new cryobiotechnological methodologies by providing microscopic images of the status of tissues and cells (e.g. amount and distribution of liquid and frozen water) within an organism at different stages of a freeze-thaw cycle.

The technique would also be an excellent aid to assessing (perhaps while experimentally manipulating) the cold hardiness strategy of species and tissues; for example, the images in Fig. 3 very clearly show a huge difference in signal between −35°C where the larva is still in the supercooled state and −70°C where the larva is frozen. They also correspond with the known viability of this species which is (in the absence of inoculative freezing) remarkably high at temperatures at or above the supercooling point of about −40°C but quickly reaches zero below this temperature [16].

MR microimaging studies of frozen [36], [45], [46] or otherwise dehydrated samples [62]–[65], [66], [67] are still limited in number and scope, much like non-invasive investigations into developing organisms or single cells therein [50]. However, MR techniques already currently provide a multitude of experimental options at the different levels of biological organization. Since the necessary equipment is being installed in more and more laboratories, such investigations will certainly contribute significantly to the understanding of living systems, even more so in light of a number of further technological advances in the MR field (for review, see [68]).

Experiments at higher magnetic field strengths result in increased signal-to-noise ratios and improved peak separability [69], cryoprobes reduce the noise [70], stronger gradients open the door to higher spatial resolution [57], while sophisticated pulse sequences allow to coax ever more subtle information out of the sample [71]–[73], and parallel acquisition schemes allow the investigation of several specimens under identical or controllably varied conditions [74]–[77]. Further progress can be expected from mobile MR devices [74]–[77], remote detection schemes [78] and, though perhaps more slowly, from approaches based on nuclei other than ^1^H [79]–[82].

Like any other imaging technique, MRI can produce artefacts if theoretical assumptions underlying the image acquisition or processing are not met by the experimental conditions (for brief overviews, see [83], [84]). For structural *in vivo* MR imaging, the major cause for artefacts is motion (as in Fig. 3) by or within the object of study during image acquisition (for a discussion in human musculoskeletal imaging, see [85]). In a cryobiological context, this is much less of a concern but in case it is, special techniques (with their own sets of assumptions) exist to correct for some types of motion artefacts [86], [87] or to quantifiy motion directly (e.g. [88], [89]). Another cause for artefacts are magnetic field inhomogeneities due to a spatially or temporally inhomogeneous magnetic susceptibility of the sample (e.g. [90]). However, what might be considered an artefact in structural imaging may be of value in functional imaging–the most popular functional MRI technique (BOLD fMRI, cf. [91]) is based on the oxygenation-dependent magnetic susceptibility of haemoglobin in blood that flows through the organism in a task-dependent manner (for recent review, see [92]). It has already observed that different regions in the body of freeze-tolerant frogs freeze at different external temperatures [46], and using high-resolution techniques, this kind of functional MRI approach can be extended to investigate such cryobiological processes in more detail.

Safety concerns play a major role in the choice of imaging techniques for biomedical applications, and one of the reasons for the popularity of MR techniques is that they (in contrast, for instance, to Computed tomography; cf. [93]) are not known to have any side effects of medical relevance if cautionary measures (e.g. concerning pace makers and implants) are followed [94], [95]. For other organisms, however, the situation may be different, especially at higher field strengths [96], [97].

Since MR emphasizes different characteristics of a sample under investigation than other imaging or spectroscopic modalities do, its combination with complementary approaches–namely with optical [98]–[102], x-ray [103] and ultrasound microscopy [104], [105], positron emission tomography [106] or chromatographic techniques [107]–harbours considerable promise, especially in light of ever more sophisticated approaches to multidimensional image processing [108]–[110] and MR-based computational morphometry [111], [112].

It should be noted that the methods described here and mentioned so far are not restricted to use in animals–they can equally (or perhaps even better, due to the absence of active motion) be applied in plants (for reviews, see [44], [113]) or other environments relevant to the species of interest–be they permafrost samples [38] or supercooled cloud droplets inhabited by bacteria [39], [114], [115].

Indeed, information about the *Solidago* host of the *Epiblema* larvae in our experiments can already be derived from data sets like the one described in Fig. 3 by simply changing the image contrast. In our case, since the focus of the experiments was on insect cold hardiness, the *Solidago* stems had been cut during insect collection but many experimental designs targeting host-parasite and other environmental interactions would also be compatible with MR experiments. Similarly, comparative analyses of taxonomically close or distant species can be performed (cf. [116]), opening up multiple windows for the study of evolutionary processes–even at the population level [77], [88]–from a cryobiological perspective [117]. Aquaporins, for example, have been found to be implicated in cold hardiness [118], also in *Eurosta* [119], and can be successfully traced by MR [120].

Given that the physiological effects of any kind of dehydration are in many aspects similar [15], [121], the methodology described here may well be suitable to the study of similar processes at other non-freezing temperatures, e.g. biomineralisation. MR spectroscopic [122], [123] and imaging studies [65], [124] in bone and fossils already point in this direction.

Furthermore, it can be expected that neighbouring fields, like biodiversity studies, will continue to profit (perhaps increasingly so) from cryotechniques as a means of preservation, and the growing popularity of cryobanking as well as the improving quality of the preserved samples might eventually lead to cryobanks taking over museal functions for molecular, cellular and tissue samples [125], [126]. Curating and monitoring these will require non-invasive microscopic technologies of the kind presented here.

### Conclusions

Freezing and dehydration of biological specimens in response to cooling below the freezing point of water can effectively be monitored by Magnetic Resonance Microscopy down to about −70°C. Consequently, this non-invasive *in vivo* technique, along with the achievable spatial, temporal and spectral resolutions, provides for very interesting non-invasive methodological options to investigate cold hardiness and–by extension–dehydration, extremophiles as well as their evolutionary and ecological relationships, and to optimize cryobiotechnological procedures.

## Supporting Information

Movie S1 ^1^H NMR images of an Epiblema larva at different temperatures. Slice series from 3D MR images, corresponding to Fig. 3, acquired at 0°C (top), −20°C, −35°C and −70°C (bottom).
(16.78 MB AVI)

Movie S2 ^1^H NMR image of an Eurosta larva at −20°C, using all spectral information. Slice series from 3D MR image, corresponding to Fig. 4, acquired at −20°C.
(8.40 MB AVI)

Movie S3 ^1^H NMR image of an Eurosta larva at −20°C, based on spectral information from the water resonance. Slice series from 3D MR image, corresponding to Fig. 4, acquired at −20°C.
(8.40 MB AVI)

Movie S4 ^1^H NMR image of an Eurosta larva at −20°C, based spectral information from the hydrocarbon resonances. Slice series from 3D MR image, corresponding to Fig. 4, acquired at −20°C.
(8.40 MB AVI)

Movie S5 ^1^H NMR image of an Eurosta larva at −40°C, using all spectral information. Slice series from 3D MR image, corresponding to Fig. 4, acquired at −40°C.
(8.40 MB AVI)

Movie S6 ^1^H NMR image of an Eurosta larva at −40°C, based on spectral information from the water resonance. Slice series from 3D MR image, corresponding to Fig. 4, acquired at −40°C.
(8.40 MB AVI)

Movie S7 ^1^H NMR image of an Eurosta larva at −40°C, based on spectral information from the hydrocarbon resonances. Slice series from 3D MR image, corresponding to Fig. 4, acquired at −40°C.
(8.40 MB AVI)

Movie S8 3D model of the water and lipid distribution in a Eurosta larva. Animation of the 3D model described in Fig. 5, based on ^1^H NMR water and hydrocarbon images obtained at −20°C K. The water-based 3D model is depicted in semi-transparent brown and the model based on the fat signal in red. Note that single cells can be discerned.
(8.83 MB AVI)

Movie S9 Temperature-weighted 3D model of an Epiblema larva. Animation of the 3D model described in Fig. 6, based on 1H NMR whole-spectrum images obtained at −35°C (brown) and −70°C (red).
(6.58 MB AVI)
